# Effective Pre-Clinical Treatment of Fish Envenoming with Polyclonal Antiserum

**DOI:** 10.3390/ijms24098338

**Published:** 2023-05-06

**Authors:** Monica Lopes Ferreira, Maria Alice Pimentel Falcão, Fernanda Miriane Bruni, Vidal Haddad, Elineide Eugênio Marques, Carla Simone Seibert, Carla Lima

**Affiliations:** 1 Immunoregulation Unit of Laboratory of Applied Toxinology (CeTICs/FAPESP), Butantan Institute, São Paulo 05503-900, Brazilcarla.lima@butantan.gov.br (C.L.); 2 Faculdade de Medicina, Universidade Estadual Paulista, Botucatu 18618-689, Brazil; 3 Environmental Sciences, Campus of Palmas, Federal University of Tocantins, Palmas 77001-090, Brazil

**Keywords:** polyclonal antiserum, neutralizing antibody, fish venom, toxic effects, *Thalassophryne nattereri*, *Scorpaena plumieri*, *Cathrops spixii*, *Potamotrygon gr orbignyi*

## Abstract

Envenomation by venomous fish, although not always fatal, is capable of causing damage to homeostasis by activating the inflammatory process, with the formation of edema, excruciating pain, necrosis that is difficult to heal, as well as hemodynamic and cardiorespiratory changes. Despite the wide variety of pharmacological treatments used to manage acute symptoms, none are effective in controlling envenomation. Knowing the essential role of neutralizing polyclonal antibodies in the treatment of envenoming for other species, such as snakes, this work aimed to produce a polyclonal antiserum in mice and test its ability to neutralize the main toxic effects induced by the venoms of the main venomous Brazilian fish. We found that the antiserum recognizes the main toxins present in the different venoms of *Thalassophryne nattereri*, *Scorpaena plumieri*, *Potamotrygon gr. Orbignyi*, and *Cathorops spixii* and was effective in pre-incubation trials. In an independent test, the antiserum applied immediately to the topical application of *T. nattereri*, *P. gr orbygnyi*, and *C. spixii* venoms completely abolished the toxic effects on the microcirculation, preventing alterations such as arteriolar contraction, slowing of blood flow in postcapillary venules, venular stasis, myofibrillar hypercontraction, and increased leukocyte rolling and adherence. The edematogenic and nociceptive activities induced by these venoms were also neutralized by the immediate application of the antiserum. Importantly, the antiserum prevented the acute inflammatory response in the lungs induced by the *S. plumieri* venom. The success of antiserum containing neutralizing polyclonal antibodies in controlling the toxic effects induced by different venoms offers a new strategy for the treatment of fish envenomation in Brazil.

## 1. Introduction

Venomous fish have venom glands dispersed in venom delivery structures such as retroserrated stingers or canaliculated spines that produce complex mixtures of peptides and proteins with chemical and pharmacological properties [1,2]. Accidents can occur, among other ways, in which the glandular venom-producing integumentary sheaths that cover the spine breaks and the venom is released into the wound along with the mucus, or when the canaliculated spine penetrates the tissue and the integumentary sheath that surrounds the gland presses the venom through the duct and the venom is injected into the victim. The palm of the hands and the soles of the feet are the most commonly affected sites in humans.

Almost all families and genera of venomous fish have representatives in the seas and rivers of Brazil, including the toadfish *Thalassophryne nattereri* (*Batrachoidae*), the scorpionfish *Scorpaena plumieri* (*Scorpaenidae*), the freshwater stingray *Potamotrygon gr. orbignyi* (*Potamotrygonidae*), and the catfish *Cathorops spixii* (*Ariidae*). Hundreds of accidents are estimated per year [3,4,5,6,7] but many cases go unreported, placing these accidents in the neglected status [8].

The envenomation provoked by *T. nattereri* is characterized by local intense pain, swelling, and erythema, followed by necrosis that persists for several days and is difficult to heal [9,10]. The venoms of the *C. spixii* and *P. gr. orbignyi* induce local effects such as intense pain and soft tissue edema. The pain peaks after 30 to 60 min, may radiate centrally, and can last for 48 h [11,12]. The human envenomation by *S. plumieri* is characterized by pulmonary edema that develops within 35 min of envenomation [13]. It is important to mention that in addition to local and systemic effects such as myotoxicity, neurotoxicity, and cardiorespiratory and blood pressure changes, fish venoms are potentially capable of inducing allergic responses and anaphylaxis, as demonstrated in mice [14].

Furthermore, there are currently no therapies that directly target pain, swelling, and necrosis, the most common clinical features of fish envenomation. The use of a wide range of analgesics such as tramadol hydrochloride, carbamazepine, thiamine, inhibitors of serotonin and histamine, steroidal and non-steroidal anti-inflammatory drugs like ketoprofen, or synthetic opioid like meperidine, concomitantly with antibiotics is ineffective in treating the pain or preventing the development of necrosis and consequently the systemic effects [10,15].

Investigations in mice have identified the kallikrein–kinin cascade as the major mechanism involved in the nociception as well as the edematous response induced by the venom of *T. nattereri.* Administration of tissue kallikrein inhibitor (phenyl-acetyl-Phe-Ser-Arg-N-(2,4-dinitrophenyl)-ethylenediamine—TKI) was the only therapeutic intervention able to reduce the pain and edema, but not the necrosis induced by *T. nattereri* [16].

With the failure of therapeutic interventions, chronic necrotic wounds and allergic symptoms can be triggered in patients injured by venomous fish. Therefore, an alternative therapeutic approach to these accidents is necessary.

Neutralizing antibodies are a major component of our immune defenses and for more than a century, passive immunization with polyclonal or monoclonal antibodies has been used in the treatment and prevention of infectious diseases. The role of polyclonal antibodies in host protection against snake envenomation, preventing tissue injury, venom spread, and systemic toxic responses, has been amply demonstrated [17].

An example of the effectiveness of antisera in counteracting all clinical effects of fish envenomation comes from Australia, the only country to use an antiserum produced in horses for *Synanceia horrida* (formerly known as *S. trachynis*) and *S. verrucosa* (Scorpaenidae) species. Furthermore, there is experimental evidence that this antiserum also neutralizes the pharmacological effects of other fish venoms, particularly South Australian cobbler lionfish (*Pterois volitans*) and soldier fish (*Gymnapistes marmoratus*) [18].

Although fish envenomation is frequent in Brazil, only pre-clinical studies of neutralization with anti-venom antibodies in experimental models were performed [9,19,20,21,22], and until now there are no commercially available antisera for human victims. The objective of this work was to produce an antiserum in mice composed of polyclonal IgG antibodies against multiple toxins from the venoms of the most medically important fish species in Brazil such as *Thalassophryne nattereri*, *Scorpaena plumieri*, *Potamotrygon gr*. *orbignyi*, and *Cathorops spixii* and to test its ability to neutralize the main toxic effects induced by the venoms using two different methods (pre-incubation and independent) that are considered the gold standard tests for neutralization.

## 2. Results and Discussion

### 2.1. Immunogenicity Capacity of Fish Venoms to Produce Neutralizing IgG Antibodies

Antibodies perform several key functions during infections and envenomations, including complement recruitment, opsonization, and neutralization. Specific antibodies can form immune complexes that inactivate venom toxins and are cleared by phagocytic cells. Assurance, a key quantitative tool for decision making in drug development and in the specific context of antiserum development in the early stages of development, is often based on the levels of neutralizing antibodies produced [23,24].

To dissect the relationship between immunogenicity and neutralizing antibody levels, we determined the mean anti-venom IgG titers in the serum of mice submitted to two immunization methods, with or without adjuvant. Oil-in-water adjuvants work to emulsify antigens, which may prevent antigen sequestration by circulating antibodies, enhancing free antigen delivery to lymph nodes and helping to stimulate innate immune receptors [25].

Regarding the isotype of neutralizing antibodies, it is known that IgG (IgG1, IgG2, IgG3, and IgG4 subclasses) in the blood and extracellular fluid or mucosal IgA can be both protective. IgG1 and IgG3 are usually produced in response to proteins and are the IgG subclasses most linked to neutralizing antibody activity [26].

**Figure 1:**
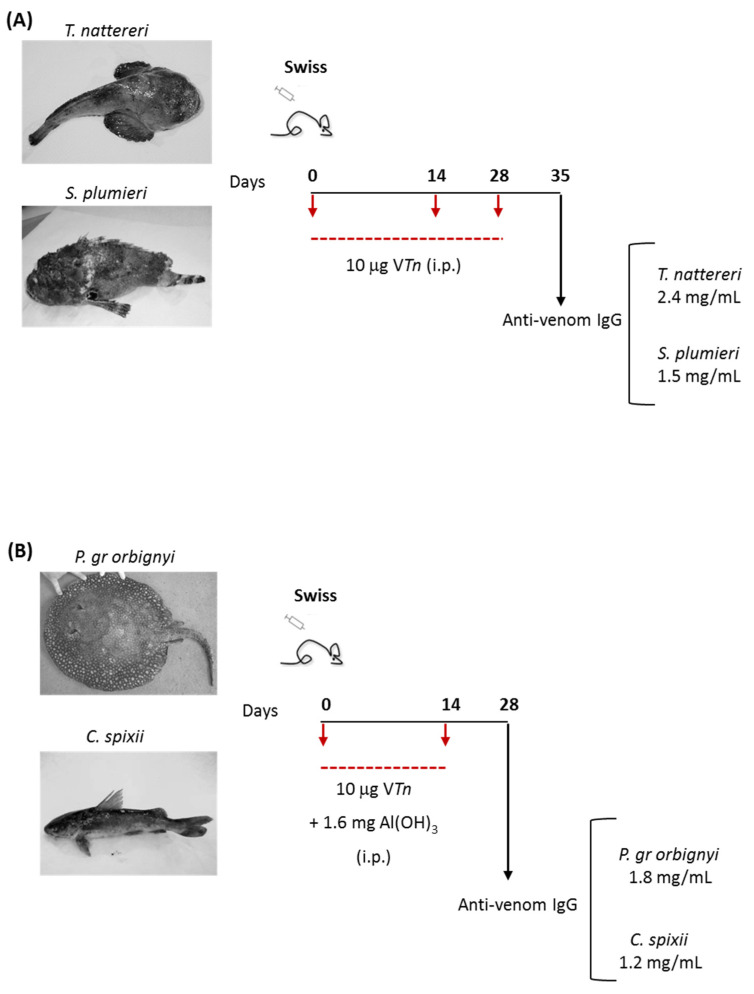
Anti-venom IgG titers in the sera of mice submitted to two immunization methods, with or without adjuvant. When mice were immunized with venoms without adjuvant, we observed that only *T. nattereri* and *S. plumieri* venoms were efficient in inducing high titers of anti-venom IgG antibodies (2.4 or 1.5 mg/mL, respectively—(**A**)). Satisfactory levels of anti-venom IgG antibodies against *P. gr orbygnyi* and *C. spixii* venoms were only achieved with the use of adjuvant (1.8 or 1.2 mg/mL, respectively—(**B**)).

When mice were immunized with venoms without adjuvant, we observed that only *T. nattereri* and *S. plumieri* venoms were efficient in inducing high titers of anti-venom IgG antibodies (2.4 and 1.5 mg/mL, respectively—Figure S1A,B and Figure 1A). On the other hand, satisfactory levels of anti-venom IgG antibodies against *P. gr orbygnyi* and *C. spixii* venoms were only achieved when venoms were emulsified with the adjuvant (1.8 and 1.2 mg/mL, respectively—Figure S1C,D and Figure 1B).

Indeed, the venoms of *Potamotrygon gr. Orbignyi* and *Cathorops spixii* showed lower immunogenicity when applied alone or even when emulsified in adjuvant (Supplementary Figure S1), demonstrating the lack of dominant epitopes involved in the humoral immunity response. It would be necessary to identify immunodominant epitopes by immunoinformatics tools associated with laboratory analysis procedures of whole venom to develop linear and discontinuous B-cell epitopes capable of eliciting neutralizing antibodies against all variants of the full-length proteins [27].

These data show that the inclusion of oil-in-water adjuvants may have an important role in generating neutralizing antibodies against *P. gr orbygnyi* and *C. spixii* venoms, but proteins from the venoms of *T. nattereri* and *S. plumieri* alone have the ability to robustly induce high antibody titers.

These results show different immunogenicities of fish venoms in the production of effective titers of neutralizing IgG antibodies, a property inherent to the immunochemical characteristics of the toxins. Lack of conformation of target epitopes and/or interference with antigen processing and presentation are possible reasons for the failure to induce adequate epitope-specific immune responses, and strategies should be considered to increase the accessibility of B-cell epitopes for inducing adequate and appropriate immunity responses.

Another way to solve this problem is the construction of polyspecific sera, capable of recognizing different regions of epitopes, obtained by joining monospecific sera induced through various immunizations.

Therefore, our strategy was to obtain a polyspecific antiserum by combining 1 mg of each monospecific serum and purifying only the IgG fraction of the serum by caprylic acid precipitation to minimize potential side effects of immunoglobulin. Our next step was to evaluate the ability of this antiserum to recognize the main toxins present in the different venoms of *T. nattereri*, *S. plumieri*, *P. gr orbygnyi*, and *C. spixii.*

**Figure 2:**
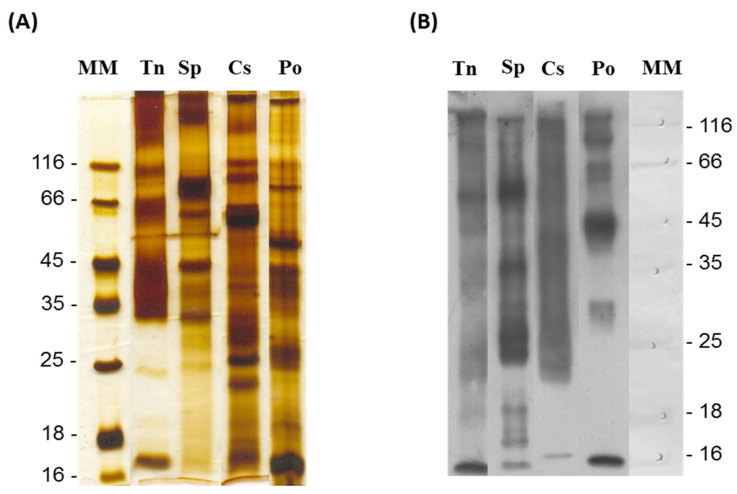
Immunochemical characterization of fish venoms and recognition by polyspecific antiserum. Venoms at 10 μg of Tn (*T. nattereri*), Sp (*S. plumieri*), Cs (*C. spixii*) and Po (*Potamotrygon gr*. *orbignyi*) venoms were separated on 4–20% SDS-PAGE gels and stained with silver (**A**) and revealed by Western blotting with the polyspecific antiserum at 10 μg/mL (**B**).

The electrophoretic profile of the venoms after running on an SDS-PAGE gel (4–20%) shown in Figure 2A reveals that the venom of *T. nattereri* is composed of the 15 kDa C-type lectin Nattectin of [28], proteins of the Natterin family around 30 to 40 kDa in size [29], and unidentified proteins around 66 kDa, 116 kDa, and above 200 kDa. The *S. plumieri* venom showed a predominance of high molecular weight bands above 116 kDa, and bands around 66 kDa, 45 kDa, and few bands below 35 kDa [30]. *C. spixii* venom presented more complex venom with a wider range of bands between 16 to- 66 kDa. The 66 kDa band was identified as the N-glycosylated WAP65-like toxin, a warm temperature acclimation-related protein. Also this venom showed bands around and above 116 kDa [31]. *P. gr orbygnyi* venom showed strong bands at approximately 16 kDa, with other bands at 25 kDa, 40 kDa, and 116 kDa [12].

All proteins in the 16 to 116 kDa bands of the four venoms were strongly recognized by the antiserum in the Western blot membrane, including bands around 25 kDa and between 18 and 25 kDa in *S. plumieri* venom that were not identified in the SDS-PAGE gel (Figure 2B).

These data show a broad neutralizing capacity of the antiserum, demonstrated by the specific interaction—with different proteins in each venoms, as well as cross-reactivity between them.

### 2.2. The Antiserum Attenuated Changes in the Microvasculature

Neutralizing antibodies could be defined as antibodies that bind to free infective particles or molecules without the capacity to bind to the host receptors. Specifically, neutralizing antibodies can reduce venom toxicity by binding to dominant epitopes of toxins, blocking their ability to binding to cellular receptors and preventing conformational changes necessary for binding with the cell membrane such as, for example, the proteins of the Natterin family that have an aerolysin domain [32] capable of forming a ring-shaped octameric pore [33] in the cell membrane and activating the inflammasome complex [34,35].

To evaluate the potential of the serum cocktail containing four cross-neutralizing antibodies to protect against acute toxic changes induced by fish venoms, we used pre-incubation and independent methods, often considered the gold standard tests for neutralization. Among the local alterations evaluated, such as edematogenic and nociceptive activities, we also included alterations in the microcirculation (arteriolar contraction, slowing of blood flow in postcapillary venules, venular stasis, myofibrillar hypercontraction, and counting of the number of rolling and adherent cells) and acute lung inflammation induced by *Scorpaena plumieri* venom.

*Thalassophryne nattereri* (niquim) are found in the northern and northeastern regions of Brazil. Experimental studies by intravital microscopy of the cremasteric microcirculation of injected-mice showed that the venom induces thrombus formation followed by complete venular and transient arteriolar stasis [36].

**Figure 3:**
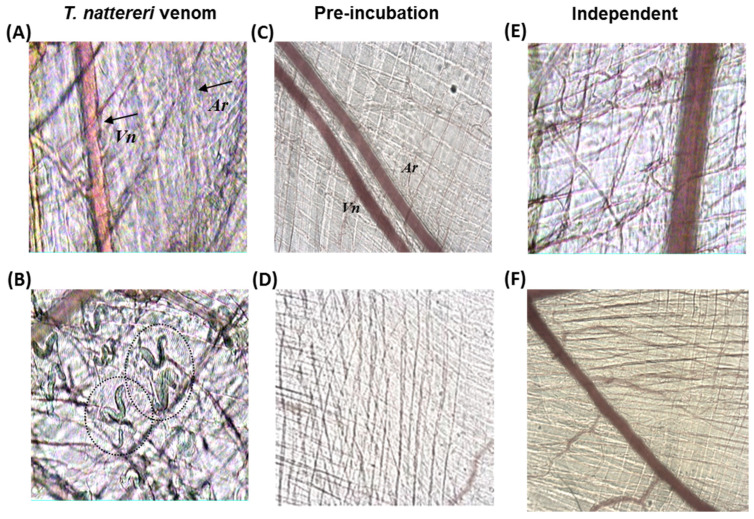
The antiserum neutralized the alterations in the microcirculation induced by *T. nattereri* venom. The topic application of venom (after 30 min) induced stasis in postcapillary venules (Vn—black arrow at left) and arteriolar constriction (Ar—black arrow at right) (**A**) and myofibrillar hypercontraction (circle—(**B**)). The pre-incubation of venom + antiserum (1:1) was able to totally neutralize the effects caused by the venom (**C**,**D**). Independent injection of antiserum, immediately (**E**) or after 15 min (**F**) of venom application were also efficient. Pictures were photographed 40 min after topical application of the mixture. Images captured with increase of 10×−1.6 optovar.

Figure 3A shows the cremasteric microcirculation of mice that had *T. nattereri* venom topically applied (30 μg venom/20 μL of sterile PBS). We observed that, 10 min after application, *T. nattereri* venom induced arteriolar contraction, with a total decrease in diameter of the vessel (Ar, black arrow at right). During this period, we also observed that the venom induced a decrease in blood flow in the venule (Vn, black arrow at left), without myofibrillar hypercontraction. However, 40 min after *T. nattereri* venom application, intense myofibrillar hypercontraction (Figure 3B), complete venular stasis, and arteriolar contraction were observed. When mice received the *T. nattereri* venom/antiserum at 1:1 ratio, we observed that after 40 min no development of any change in the caliber of arterioles or in the blood flow of the venules (Figure 3C), and no myofibrillar hypercontraction was induced (Figure 3D), reflecting a total neutralization of the effects of *T. nattereri* venom on microcirculation physiology.

In addition, the antiserum (1:2 ratio) intravenously applied immediately (Figure 3E) or after 15 min of topic *T. nattereri* venom application (Figure 3F), completely abolished the toxic effects induced by *T. nattereri* venom in the arterioles, venules, and myofibrils.

*Cathorops spixii* (catfish) is widely distributed on the coast of Brazil. Intravital analysis of the inflammatory reaction induced in the microcirculation of mice by its venom demonstrated that both mucus and sting venoms induce large numbers of rolling and adherent leukocytes in the postcapillary venules of the cremaster muscles [11].

**Figure 4:**
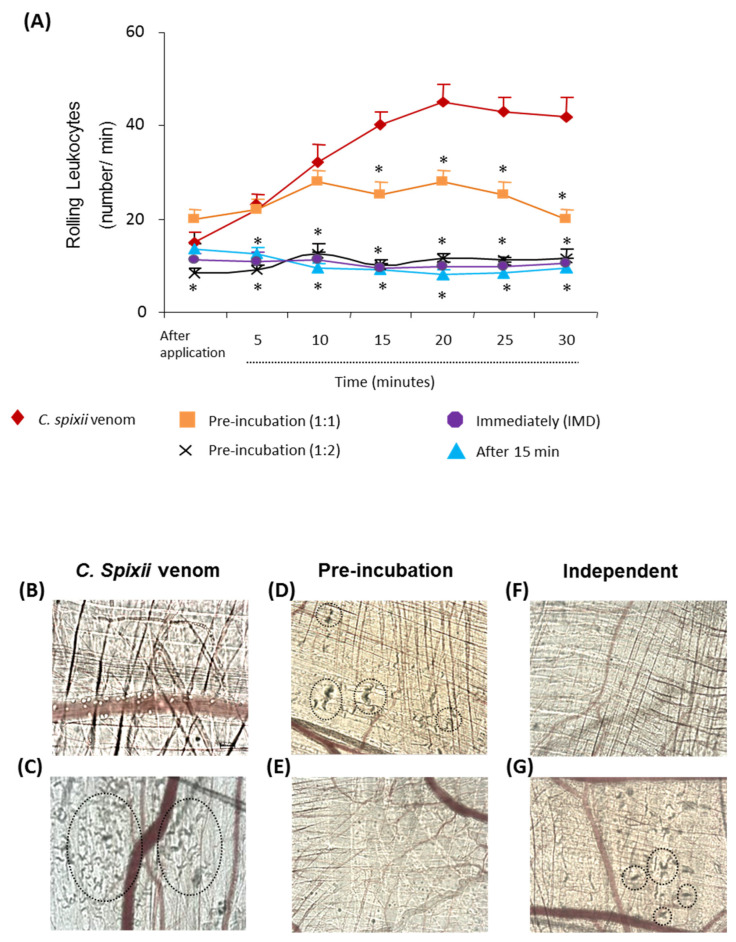
The antiserum neutralized the alterations in the microcirculation induced by *C. spixii* venom. The topical application of venom induced alterations in postcapillary venules such as an increase in rolling leukocytes (**A**,**B**) and injury to muscle fibers (**C**). All treatments were effective in neutralizing the recruitment of leukocytes (**A**,**B**). * *p* < 0.05 compared with venom-injected mice. Myofibrillar hypercontraction (**C**) was neutralized by antiserum at a 1:2 ratio (**E**) but not at 1:1 (**D**). The antiserum at a 1:2 ratio administered immediately (**F**) prevented the appearance of myofibrillar hypercontraction, but when apllied after 15 min, the antiserum did not have any neutralizing capacity (**G**). Pictures were photographed 40 min after topical application of the mixture and are representative of observations in three mice. Images captured with increase of 10×−1.6 optovar.

As shown in Figure 4, the *C. spixii* venom induced an increased number of rolling leukocytes in postcapillary venules (Figure 4A,B) 10 min after its topical application, which remained high for up to 30 min. Next, the neutralizing capacity of the antiserum was evaluated and we observed that the pre-incubation treatment at a ratio of 1:1 reduced the number of rolling leukocytes in the postcapillary venules by—43% after 15 min and remained reduced for up to 30 min. On the contrary, the mixture at a 1:2 ratio in pre-incubation assay or the application of the antiserum immediately or 15 min later completely inhibited the high number of rolling leukocytes induced by the venom (Figure 4A).

*C. spixii* venom also caused intense myofibrillar hypercontraction, after 40 min (Figure 4C). The pre-incubated venom–antiserum mixture at a ratio of 1:1 partially neutralized the myofibrillar hypercontraction (Figure 4D), but at a 1:2 ratio, these effects were completely inhibited (Figure 4E). Treatment with the antiserum at a 1:2 ratio immediately (Figure 4F) after *C. spixii* venom application completely prevented the toxic effects on the muscle fibers, reducing myofibrillar hypercontraction. However, the intravenous application of the antiserum after 15 min was not able to abolish the venom’s ability to induce myofibrillar hypercontraction (Figure 4G).

In Brazil, freshwater stingrays are very common in the northern, central-western, and southeastern regions. The family Potamotrygonidae is endemic to the fluvial systems of South America [37]. Intravital microscopy of the cremasteric microcirculation of mice demonstrated an intense inflammatory reaction induced by the venom of *P. gr. Orbygnyi*, characterized by increased number of leukocytes rolling along the endothelium [12].

**Figure 5:**
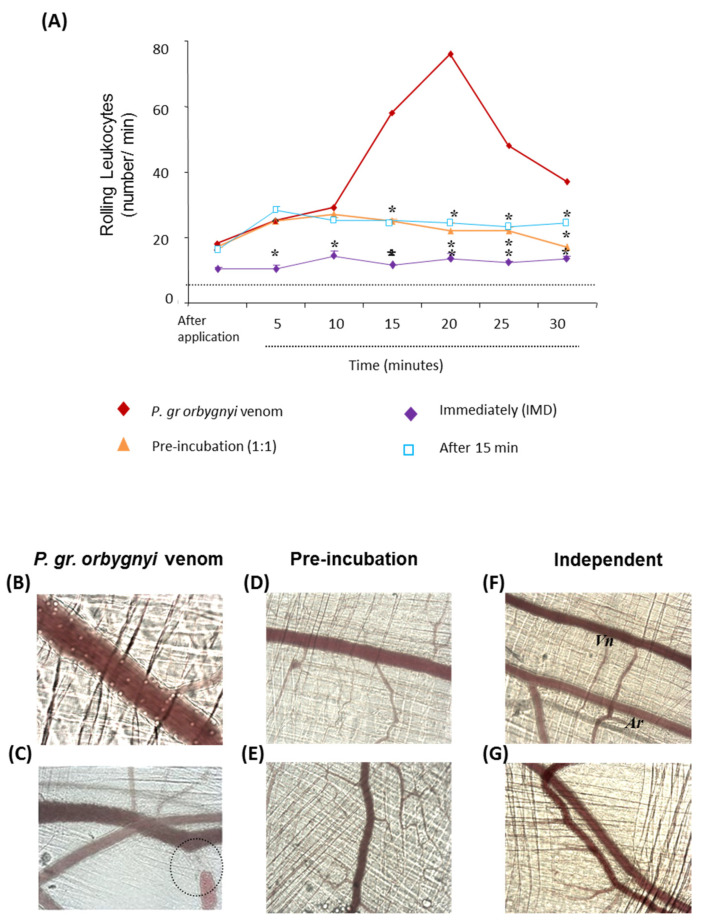
Neutralization of toxic effects induced by *P. gr orbygnyi* venom by intravital microscopy. The topical application of venom induced an increase in rolling leukocytes (**A**,**B**) in the first minutes, following prominent thrombus formation in venules (**C**). The antiserum in pre-incubation (**D**,**E**) or applied immediately (**F**) or after 15 min (**G**) was efficient in neutralizing both the increased recruitment of leukocytes as well as the alterations in venules. * *p* < 0.05 compared with venom-injected mice. Pictures were photographed 40 min after topical application of the mixture and are representative of observations in three mice. Images captured with increase of 10×−1.6 optovar.

As we can see in Figure 5, the venom of *P. gr. orbygnyi* also induced changes in postcapillary venules by increasing the number of rolling leukocytes from 15 min to 30 min, with a peak at 20 min (Figure 5A,B). All treatments (topical application of the mixture at a 1:1 or 1:2 ratio or intravenous application of the antiserum immediately or 15 min later) completely blocked the increased rolling leukocytes induced by the venom of *P. gr. orbygnyi*. Also, thrombi induced in the venules within 40 min (Figure 5C) and total stasis of blood flow in the small caliber venules were completely abolished by all treatments [topical application of the mixture at a 1:1 (Figure 5D) or 1:2 (Figure 5E) ratio, and intravenous application of the antiserum at 1:2 ratio immediately (Figure 5F) or 15 min later (Figure 5G)].

These results show that high levels of neutralizing IgG antibodies in the antiserum provide protection against toxic effects in the microcirculation, mainly in the prevention of leukocyte rolling in the postcapillary venules, alteration in the diameter of arterioles, and decrease in blood flow in the venules, without controlling *C. spixii*-venom-induced myofibrillar hypercontraction. Furthermore, we observed that the effective control of these alterations in the microcirculation requires the application of the antiserum immediately after envenomation.

### 2.3. The Antiserum Prevented Edema and Nociception

**Figure 6:**
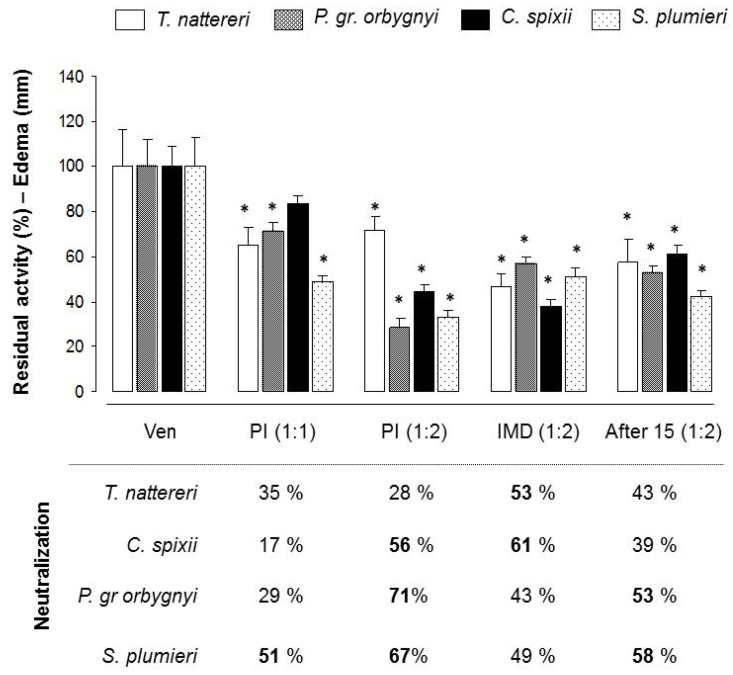
Neutralization of edematogenic activity induced by *T. nattereri*, *C. spixii*, *S. plumieri*, and *P. gr orbygnyi* venoms by antiserum. The pre-incubated mixtures of each venom (30 μg/animal) with 30 μg (1:1) or 60 μg (1:2) of antiserum was injected into the intraplantar region of the right hind footpad of mice. Local edema was quantified by measuring the thickness (mm) of injected paws with a paquimeter 30 min after injection. For the independent administration of antiserum, mice were treatment i.v. immediately (IMD) and 15 after the injection of each venom. * *p* < 0.05 compared with venom-injected mice. The efficacy of the antiserum was reported as the concentration of antibody required to reduce venom-induced toxic activities by 50%.

The alterations in capillary hemodynamics favor the movement of fluids from the vascular space into the interstitium, culminating in edema formation [38]. With the alterations induced in the microcirculation by the venoms, the next step was to verify the effect of the neutralizing antibodies in controlling the edema. The edema was quantified by measuring the thickness (mm) of injected paws 30 min after venom injection. The efficacy of neutralizing antibodies was reported as the concentration of antibody required to reduce the venom-induced toxic activities by 50% (Figure 6).

The antiserum in a 1:1 ratio provided 51% protection against edema formation only for the *S. plumieri* venom in the pre-incubation assay. When the ratio was increased to 1:2, we observed that this pre-incubation method produced an effective reduction of 56%, 67%, and 71% in edema induced by *C. spixii*, *S. plumieri*, and *P. gr orbygnyi* venoms, respectively, demonstrating a dose–response relationship.

The independent method treatment showed that immediate injection of antiserum at a 1:2 ratio was effective in reducing edema induced by *T. nattereri* and *C. spixii* venoms by 53% and 61%, respectively. However, application after 15 min only reduced edema induced by *P. gr orbygnyi* and *S. plumieri* venoms by 53% and 58%, respectively. Protection against *T. nattereri*-venom-induced edema provided by immediate treatment decreased from 53% to 43% when injected after 15 min. The same decrease was seen for *C. spixii*-venom-induced edema, from 61% to 39%. On the contrary, even when injected after 15 min, the antiserum was able to neutralize the edema induced by the *P. gr orbygnyi* and *S. plumieri* venoms.

Lopes-Ferreira et al. [16] identified the kallikrein–kinin cascade as the major mechanism involved in the pain nociception as well as the edematous response induced by the venom of *T. nattereri.* As for *C. spixii* venom, Ramos et al. [31] demonstrated differences regarding the action of the peptides and proteins present in the venom sting. While the protein fractions produced a typical inflammatory process in post-capillary venules, the peptide fractions caused more harmful effects, such as changes in blood flow, venular stasis, hemorrhages, and changes in the arteriolar wall diameter. In addition, Lopes-Ferreira et al. [39] found that serotonin (5-hydroxytryptamine—5-HT), leukotriene, and prostaglandin are involved in the edematogenic and nociceptive responses induced by *Pseudoplatystoma fasciatum*.

These data confirm the different signaling pathways involved in the induction and kinetics of edema by fish venoms, and also point to the action of preformed molecules, quickly released by the action of *T. nattereri* and *C. spixii* venoms as responsible for the vasodilator effect leading to plasma extravasation.

Pain results from the action of inflammatory mediators (adenosine, bradykinin, 5-HT, and prostanoids, among others) on peripheral sensory neurons and central sites in the spinal cord and brain. Furthermore, leukocytes recruited and activated in the inflammatory response release additional pain-inducing agents that act by modifying the response properties of primary afferent nociceptors and their activation/regulation by subsequent stimuli. Moreover, tissue inflammation also influences the central processing of nociceptive inputs in the dorsal horn of the spinal cord [40].

**Figure 7:**
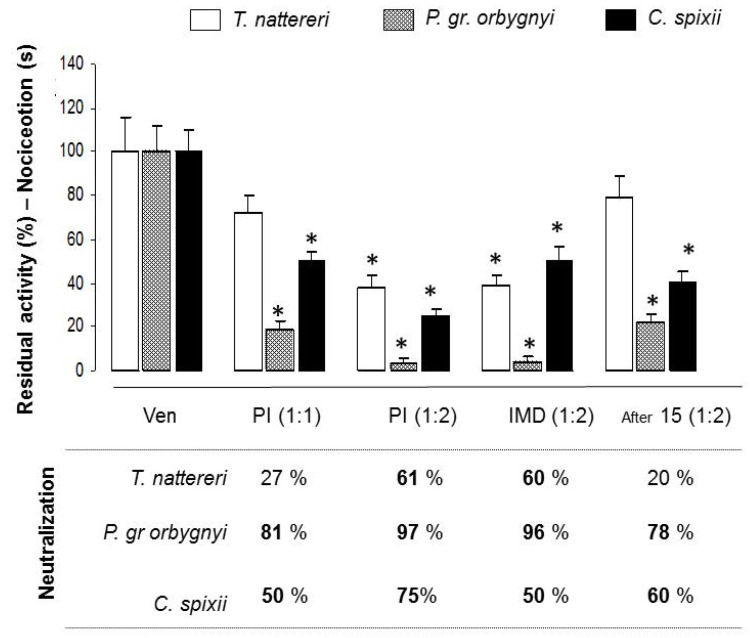
Neutralization of the pain nociception activity induced by *T. nattereri*, *C. spixii*, and *P. gr orbygnyi* venoms by antiserum. Groups of five mice were injected intraplantarly with pre-incubated mixtures of each venom (30 μg/animal) with 30 μg (1:1) or 60 μg (1:2) of antiserum. Mice were treated by i.v. injection of antiserum immediately (IMD) or 15 after the injection of each venom in the paw and nociception was evaluated. * *p* < 0.05 compared with venom-injected mice. The efficacy of antiserum was reported as the concentration of antibody required to reduce venom-induced toxic activities by 50%.

The evaluation of nociception protection by the antiserum is demonstrated in Figure 7. Compared to the protection provided by the antiserum against edema (mean protection of 59%), we observed that the neutralization of pain was more efficient, with a mean protection of 71%. The treatment methods presented a range between 50 and 97% efficiency.

Pre-incubation at a 1:1 ratio ensured protection against the pain induced by *P. gr orbygnyi* and *C. spixii* venoms by 81% and 50%, respectively, which increased to 97% and 75% when the ratio was 1:2. Furthermore, the pain induced by *T. nattereri* venom that was not controlled by the 1:1 ratio was minimized by 61% at a 1:2 ratio.

Immediate injection of antiserum controlled the pain induced by *T. nattereri* (60%), *P. gr orbygnyi* (96%), and *C. spixii* (50%) venoms. However, when treatment was delayed by 15 min, the efficacy was lost for *T. nattereri* (20%), and dropped to 78% and 60% for *P. gr orbygnyi* and *C. spixii* venoms, respectively.

These results show that nociception induced by *T. nattereri*, *P. gr orbygnyi*, and *C. spixii* venoms is more efficiently controlled by antiserum neutralizing antibodies compared with edematogenic activity control, and that the delay in applying the treatment reduces the effectiveness of neutralization of the edematogenic activity induced by *T. nattereri* and *C. spixii* venoms and the nociception induced by *T. nattereri* venom.

### 2.4. The Antiserum Neutralizes Acute Lung Inflammation

Members of the genus *Scorpaena* are found on the Brazilian coast, with *S. plumieri* (mangangá) being the most abundant species. Mouse models recapitulate the cardiotoxic and hypotensive effects observed in humans [41]. When mice are injected in the footpad or peritoneal cavity, the venom recirculates and reaches the airways, promoting an acute inflammatory response in the lungs [30]. Thus, our next step was to evaluate the ability of the antiserum to neutralize this systemic response.

**Figure 8:**
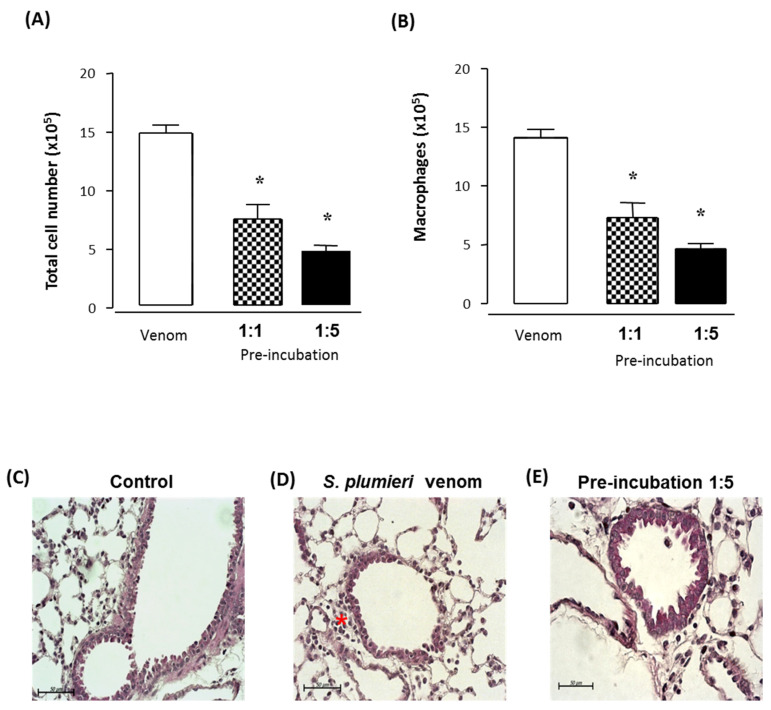
Neutralization of the acute lung injury induced by *Scorpaena plumieri* venom by antiserum. Groups of mice were intraplantarly injected with 100 μg of *S. plumieri* venom diluted in 30 μL sterile PBS or pre-incubated mixtures of *S. plumieri* venom/antiserum at 1:1 or 1:5 ratios. After 24 h, mice were euthanized and BAL was collected for total leukocytes counts (×10^5^, (**A**)) and characterized mainly for accumulation of macrophages (×10^5^, (**B**)). H&E staining of lung tissues from venom-injected mice showed peribronchiolar infiltration of mononuclear cells in mice injected with venom (**D**) compared to control mice that showed no inflammatory infiltrate (**C**). Panel (**E**) illustrates the airways of control mice, injected with the venom and those treated with the antiserum. * *p* < 0.05 compared with venom-injected mice.

In Figure 8A, we observed that total cell recruitment to the BAL 24 h after venom injection into the mice’s paw was blocked in the pre-incubation assay with a 1:1 (50%) or 1:5 (66%) ratio, as well as the number of macrophages (46% and 65% decrease, respectively—Figure 8B). Interstitial cellular infiltration was evaluated in H&E-stained sections from mice treated with the supernatant of the venom/antiserum mixture at a 1:5 ratio. We observed peribronchiolar infiltration of mononuclear cells in mice injected with *S. plumieri* venom (Figure 8D) compared to control mice that showed no inflammatory infiltrate (Figure 8C). Figure 8E illustrates the absence of peribronchiolar infiltrate in the mice treated with the antiserum in the pre-incubation assay at a 1:5 ratio.

## 3. Materials and Methods

### 3.1. Mice

Female, 7–8-week-old Swiss wild-type mice were obtained from a colony at Butantan Institute. The mice were maintained in sterile microisolators with sterile rodent feed and acidified water, and were housed in positive-pressure air-conditioned units (25 °C, 50% relative humidity) on a 12 h light/dark cycle. The experiments were carried out under the laws of the National Council for Animal Experiment Control (CONCEA) and approved by the Butantan Institute’s Animal Use Ethics Commission (Permit Number: 379/07).

### 3.2. Fish and Venoms

Adult specimens of both sexes of *T. nattereri*, *P. gr orbignyi*, *C. spixii*, and *S*. *plumieri* were collected in the Brazilian state of Alagoas, Tocantins, and São Paulo. Authorization (14693-1 and 45407-1) was approved by the IBAMA (Instituto Brasileiro do Meio Ambiente e dos Recursos Naturais Renováveis). Venoms were obtained using different process of extraction [14,30,31,42] and were immediately frozen at −20 °C until use. Protein content was determined by the colorimetric method of Bradford, 1976 [43], using bovine serum albumin (Sigma) as the standard. Endotoxin content was evaluated (resulting in a total dose < 0.8 pg LPS) with a QCL-1000 chromogenic Limulus amoebocyte lysate assay (Bio-Whittaker, Walkersville, MD, USA) according to the manufacturer’s instructions. The electrophoretic profile of the venoms was evaluated using 10 μg of each in a 4–20% SDS-PAGE acrylamide gradient under non-reducing conditions.

### 3.3. Immunization Protocols for Obtaining Venom Monospecific Serum

To obtain the individual venom-specific sera, groups of mice (*n* = 10/group) were immunized intraperitoneally (i.p.) with 10 μg of each separated venom diluted in 500 μL of sterile saline. After 14 and 28 days, mice were boosted with the same dose of venom. On day 35, mice were killed and bled by cardiac puncture. Alternatively, independent groups of mice (*n* = 10/group) were i.p. injected with 10 μg venom emulsified in 1.6 mg Al(OH)_3_ as adjuvant on day 0. Mice were challenged with venoms (10 μg/animal, i.p.) on day 14 and bled by cardiac puncture on day 28 (Figure 1). Each serum individually had its venom-specific IgG content determined by ELISA (Supplementary Figure S1) using plates covered with venoms at 1 μg/mL; each monospecific serum at 1:40 was compared with a standard curve using known concentrations of purified IgG murine antibody (0107-01, Southern Biotech, Birmingham, AL, USA). The protocol capable of generating higher IgG titers was chosen to obtain monospecific antisera. Then, the specificity of each monospecific antiserum was evaluated by Western blot (WB, Supplementary Figure S2) using venom samples at 10 μg and each antiserum at 1:20 (anti-*T. nattereri* venom at 120 μg/mL, anti-*P. gr orbignyi* venom at 90 μg/mL, anti-*C. spixii* venom at 60 μg/mL, and anti-*S*. *plumieri* venom at 75 μg/mL).

### 3.4. Purification of Antiserum IgG Fraction

A volume containing 1 mg of each monospecific antiserum was mixed to compose the polyspecific antiserum. For purification of the IgG fraction, the polyspecific antiserum was precipitated using caprylic acid, according to Kurtović et al. [44]. The efficiency of the fractionation was assayed by 8% SDS-PAGE gel (Supplementary Figure S3). Peptide mass fingerprint analysis and liquid chromatography coupled to tandem mass spectrometer (LC-MS/MS) confirmed the identity of mouse IgGs in the peptides generated after trypsinization of the band with a molecular mass above 116 kDa obtained from the fraction purified with caprylic acid (Supplementary Table S1). The IgG fraction of the polyspecific antiserum (called from now antiserum) was evaluated for its ability to recognize all venoms by WB at a dilution of 1:100 (10 μg/mL) (Figure 2).

### 3.5. Methods for Studying the Neutralization Capacity of Antiserum

Since none of these venoms have a lethal effect, the ability of the antiserum to neutralize the acute toxic alterations was evaluated using pre-incubation and independent methods. For the (1) pre-incubation method, the supernatant obtained after centrifuging the mixture (pre-incubated at 37 °C for 30 min) of venoms at 30 μg + antiserum at 30 μg or 60 μg (1:1 or 1:2) was applied topically or intravenously in mice; and (2) the independent method, the antiserum at 60 μg was applied intravenously in mice immediately or after 15 min of the injection of each venom (30 μg) at ratio of 1:2. In the pre-incubation method for neutralization of acute lung inflammation induced by *S. plumieri* venom, we used 100 μg venom with 100 μg (1:1) or 500 μg (1:5) of antiserum. Normal mouse serum substituted the antiserum in control group of mice.

#### 3.5.1. Evaluation of Microcirculation Alterations in Cremaster Muscle

Mice (*n* = 4–7) were anesthetized by an i.p. injection of 2% xylasine (Calmiun^®^, Agener União, São Paulo, SP, Brazil) and with 0.5 g/kg of ketamine (Holliday-Scott SA, Buenos Aires, Argentina). The scrotum was opened and the cremaster muscle was exteriorized. After longitudinal incision with a cautery and spreading of the muscle over a cover glass, the epididymis and testis were mobilized and pinned aside leading to full microscopic access to the cremaster muscle microcirculation. The exposed tissue was superfused with bicarbonate-buffered saline, pH 7.4, warmed to 37 °C. The postcapillary venules, with a diameter of 25–40 μm, were chosen and the interaction of leukocytes with the luminal surface of the venular endothelium was evaluated by counting the number of rolling leukocytes every 10 min after application of each venom for 30 min. Rolling leukocytes were defined as those moving at a velocity less than erythrocytes (reduced to approx. 5 μm/s) and demonstrated a clear rolling motion. The number of adherent cells was expressed as the number per 100 μm length of venule. The cremaster muscles of the mice were exposed to a topic application of 20 μL of 30 μg venoms in sterile PBS. The negative control mice were submitted to topic application of sterile PBS. Intravital microscopy was conducted for 40 min on an upright microscope (Axiolab, Carl Zeiss, Oberkochen, Germany) with a saline immersion objective (SW40/0.75 numerical aperture, Zeiss, Jena, Germany) coupled to a photographic camera (AxioCam Icc1, Carl Zeiss, Oberkochen, Germany) using a 10/0.3 longitudinal distance objective/numeric operture and 1.6 optovar (Carl Zeiss, Oberkochen, Germany).

#### 3.5.2. Nociceptive Activity

For nociceptive tests, each mouse (*n* = 4–7) was kept in an adapted chamber mounted on a mirror. After a 10 min adaptation period, the mice were injected with venoms (30 μg of each venom/animal) in a fixed volume of PBS (30 μL) into the right footpad. The control group was injected with sterile PBS. The mice were then returned to the observation chamber and the amount of time spent licking or biting each hind paw was recorded for 30 min and taken as the index of nociception.

#### 3.5.3. Edema-Induced Activity

Samples of 30 μL containing 30 μg of venoms were injected into the right footpad of mice (*n* = 4–7). Local edema was quantified by measuring the thickness of injected paws with a paquimeter (Mytutoyo, Kawasaki, Japan) 30 min after venom injection. Mice injected with 30 μL of sterile PBS were considered as control group. The results were presented by the difference between experimental and control footpad thicknesses.

#### 3.5.4. Acute Lung Inflammation Induced by *Scorpaena plumieri* Venom

*S. plumieri* venom was injected in the intraplantar region of the hind foot paw of mice (*n* = 4–7) according to Boletini-Santos et al. [30]. After 24 h, mice were euthanized with an i.p. pentobarbital injection (60 mg/kg; Sanofi, Libourne, France), the chest wall was opened, and the tracheas were cannulated to obtain bronchoalveolar lavage fluid (BAL). The collected BAL was centrifuged (170× *g* for 10 min at 20 °C), and the resulting cell pellet was then resuspended in 1 mL of PBS. Cell counts were performed using a hemocytometer, and cytocentrifuge (Cytospin II; Shandon, Cheshire, UK) slides were stained (Hema 3, Scientific Products, Chicago, IL, USA). For differential cell counts, 300 leukocytes were enumerated and identified as macrophages on the basis of staining and morphologic characteristics using a conventional light microscope (Axio Imager A1, Carl Zeiss, Germany). Paraffin-embedded sections of the left lung were stained with hematoxylin and eosin (H&E). All slides were examined with light microscopy at a magnification of 10 or 40× (Axio Imager A1, Carl Zeiss, Germany).

### 3.6. Statistical Analysis 

All values were expressed as the mean ± SEM of three independent experiments. Parametric data were evaluated using analysis of variance, followed by the Bonferroni test for multiple comparisons. Non-parametric data were assessed using the Mann–Whitney test. Differences were considered statistically significant at *p* < 0.05. The GraphPad Prisma 6 (Graph Pad Software, v6.02, 2013) statistical package was employed.

## 4. Conclusions

There are currently no therapies that directly target edema, pain, and local tissue necrosis, the most common clinical features of fish envenoming in Brazil. In this study, we generated a polyspecific antiserum made with venoms from the most medically important species of fish in Brazil (*Thalassophryne nattereri*, *Scorpaena plumieri*, *Potamotrygon* gr. *orbignyi,* and *Cathorops spixii*) and used in vivo assays, considered the gold standard test for neutralization, to evaluate the neutralizing antibody efficacy against acute toxic injuries, including alterations in microcirculation (arteriolar contraction, slowing of blood flow in postcapillary venules, venular stasis, myofibrillar hypercontraction, and number of rolling and adherent cells), edematogenic and nociceptive activities, and lung inflammation.

We demonstrated that an antiserum cocktail composed of polyclonal IgG antibodies against multiple toxins from the four fish venoms was efficient in protecting mice against toxic effects when applied at a 1:2 or 1:5 ratio in pre-incubation or immediately after the accident. The antiserum blocks the complex responses induced by all venoms, such as alterations in the microcirculation, edema, pain, and leukocyte infiltration into the lungs of mice. Another clinically relevant dimension of the dose–response curve data indicate that the therapeutic benefits of the antiserum even after 15 min of envenomation might be improved by increasing the concentration of IgG in the antiserum.

The successes of this study offer a new strategy to treat fish envenoming in Brazil providing optimal protection against the toxic effects typical of each species and provide hope for the development of a universal polyspecific antiserum effective in treating stings from venomous fish.

However, it is important to keep in mind that no matter how precisely we can measure the effects of neutralizing antibodies in in vivo mouse models, these animal models remain imperfect mimics of human envenomation and require that correlates of protection can be established.

Together, our results demonstrating the effectiveness of the polyspecific antiserum reinforce its application as a therapy capable of reversing the effects of envenoming by fish in Brazil.

## Data Availability

Not applicable.

## Supplementary Materials

The supporting information can be downloaded at: https://www.mdpi.com/article/10.3390/ijms24098338/s1.
