# Supplementary material for: Effective Pre-Clinical Treatment of Fish Envenoming with Polyclonal Antiserum
Source: Int J Mol Sci. 2023 May 6;24(9):8338. doi: 10.3390/ijms24098338 (PMC10179662; doi:10.3390/ijms24098338)
Supplement: Supplementary file 1 [file ijms-24-08338-s001.zip › Supplementary Figs and Table ijms-2279597.pdf]

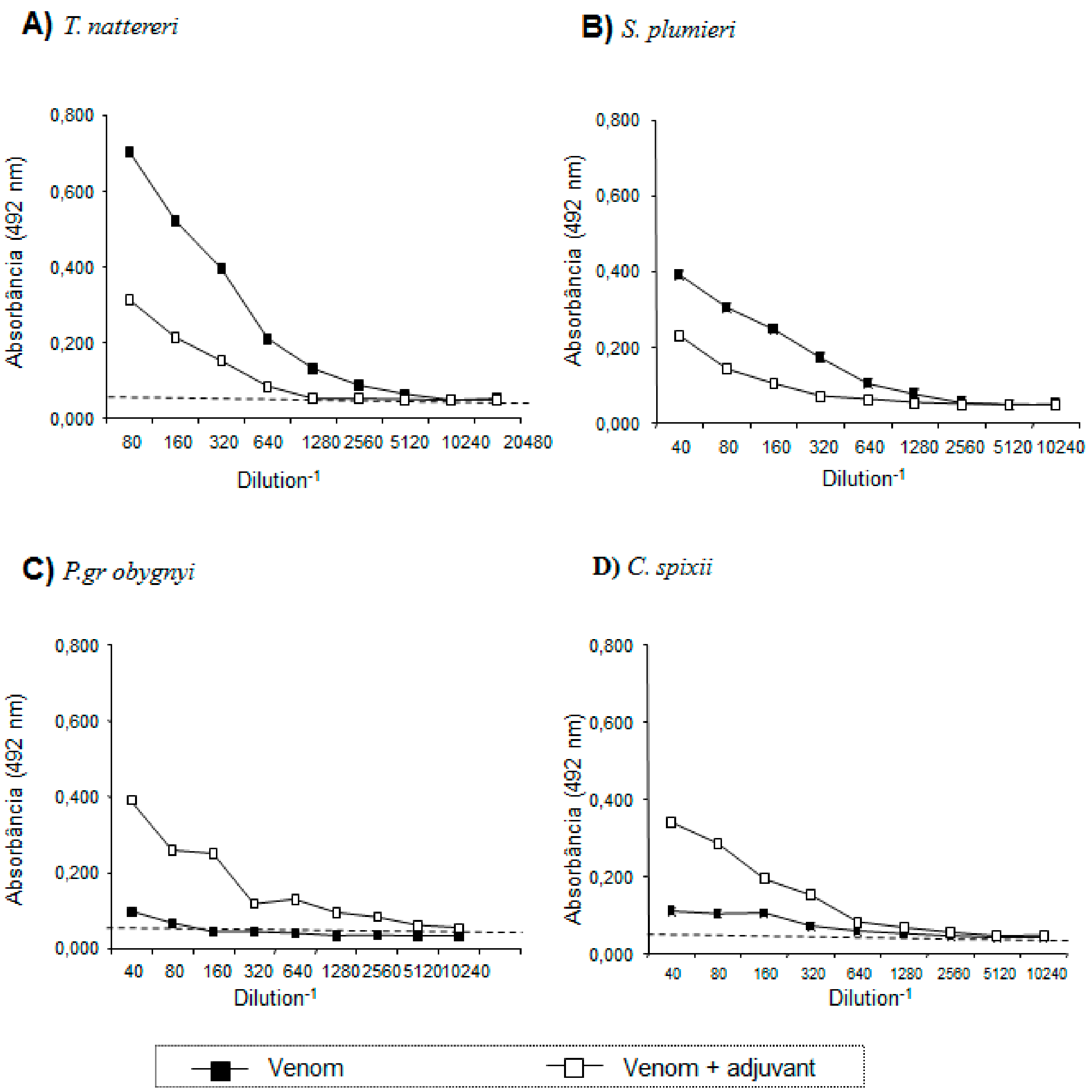

Figure S1. Comparison of the effectiveness of immunization using venoms without or with adjuvant. To obtain the specific sera, mice were immunized intraperitoneally (i.p.) with 10 ug of each venom separately diluted in 500 uL of sterile saline solution. After 14 and 28 days, they received the same dose of venom. On day 35, mice were killed and exsanguinated by cardiac puncture. Alternatively, independent groups of mice were injected i.p. with 10 ug of each venom emulsified in 1.6mg Al(OH)3 as adjuvant on day 0. On day 14, they were challenged with the venoms alone (10 ug/animal, i.p.). On the 28th they were killed and bled by cardiac puncture. Each serum was individually tested for venom-specific IgG content determined by ELISA (Supp. Figure S1) using plates coated with each venom at 1 ug/ml, which were added from each monospecific serum at 1:40 and compared with the standard curve of purified murine IgG antibody.

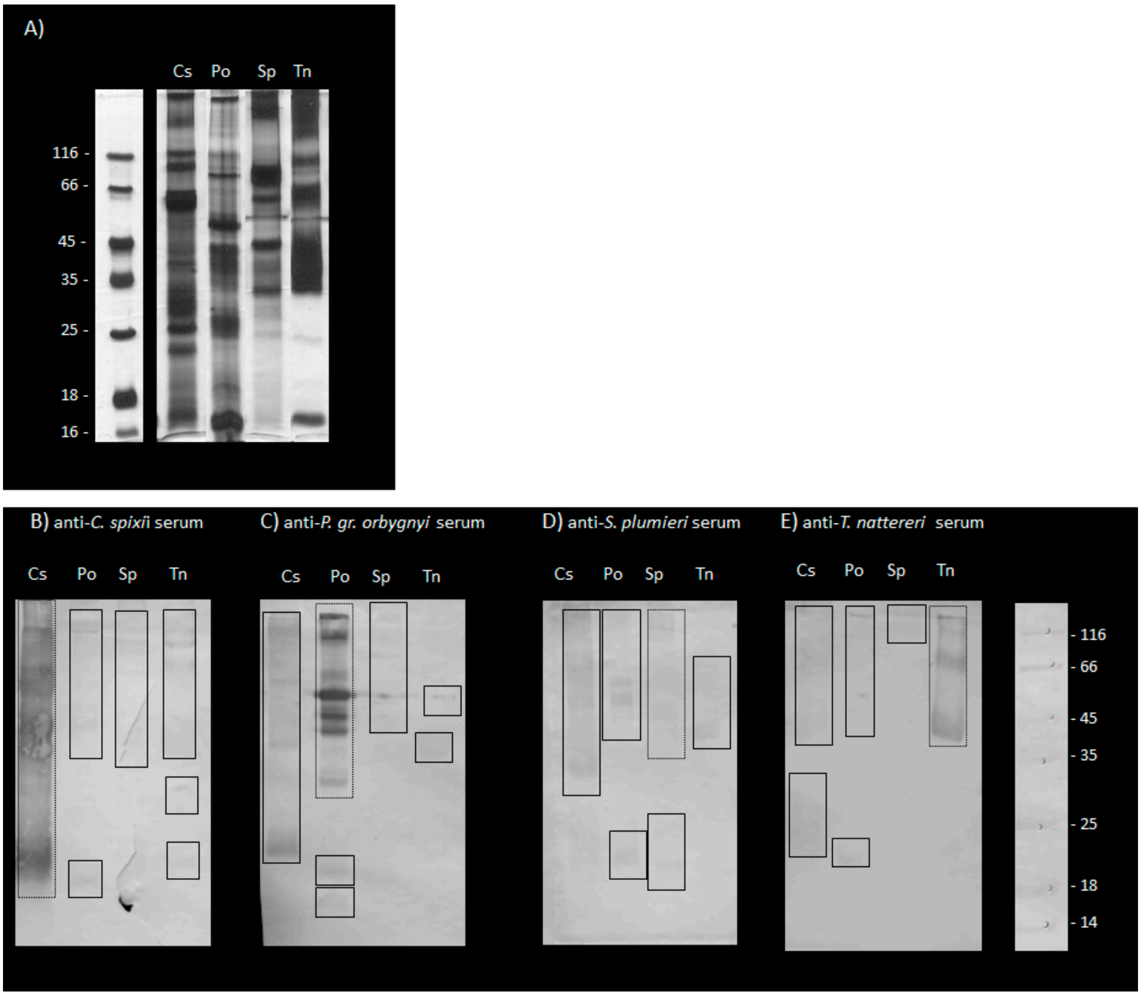

Figure S2. Identification of different venom proteins by monospecific anti-serum. The venoms (10 g) of *C. spixii* (Ca), *P. gr orbygnyi* (Po), *S. plumieri* (Sp), and *T. nattereri* (Tn) were fractionated by SDS-PAGE (A) and then transferred to nitrocellulose membranes that were developed with the monospecific sera of each venom (B-E). The numbers on the left and right indicate the molecular mass markers.

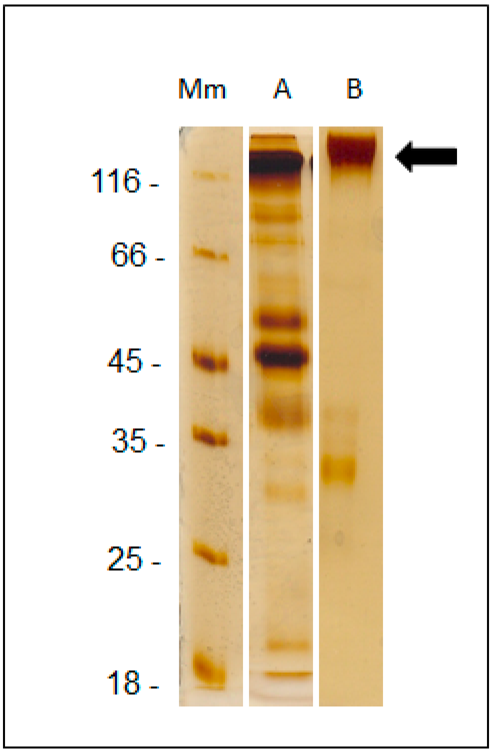

Figure S3. Electrophoretic profile of polyspecific serum after caprylic acid fractionation. Aliquots of 10 ul of polyspecific serum before (A) and after fractionation by caprylic acid (B) were subjected to SDS-PAGE electrophoresis under non-reducing conditions on an 8% polyacrylamide gel and subsequently stained with silver. The numbers on the left indicate molecular mass markers (b-galactose 116 kDa; bovine serum albumin 66.2 kDa; ovalbumin 45 kDa; lactate dehydronegase 35 kDa; restriction endonuclease Bsp98I 25 kDa, b-lactoglobulin 18.4 kDa). The arrow indicates the IgG fraction band subjected to trypsin digestion (peptide mass fingerprint).

Table S1. Identification of the peptides in the 116 kDa band of the IgG fraction from mice (*Mus musculus*) after digestion with trypsin

Me	Mc	Peptides (N-terminal)
1099.65	1099.65	K*DVLTITLTPK*V
1143.51	1143.52	K.SSSTAYMELR*S
1209.66	1209.68	K.APQVYTIPPPK.E
1242.61	1242.66	R.VNSAAFPAPIEK.T
1620.68	1620.71	R.SLTSEDSAVYYCAR.G
1796.90	1796.95	K.APQVYTIPPPKEQMAK.D
1812.91	1812.94	K.APQVYTIPPPKEQMAK.D
1852.89	1852.91	R.SVSELPIMHQDWLNGK.E
1978.89	1978.91	K.NTQPIMNTNGSYFVYSK.L
2257.09	2257.12	R.SVSELPIMHQDWLNGKEFK.C
2273.06	2273.11	R.SVSELPIMHQDWLNGKEFK.C
2875.41	2875.45	K.TTPPSVYPLAPGSAAQTNSMVTLGCLVK.G
2921.33	2921.39	R.DCGCKPCICTVPEVSSVFIFPPKPK.D

Me. Expected mass; Mc, calculated mass; * Trypsin cleavage sites

To verify the purification of IgG fraction in polyspecific serum after treatment with caprylic acid, we used Peptide mass fingerprint and Liquid chromatography coupled to tandem mass spectrometer (LC-MS/MS) methodologies. In Figure S1, we observe that treatment with caprylic acid allowed the purification of IgG fraction with high molecular mass, above 116 kDa, which were confirmed by LC-MS/MS analysis. The sequences of the peptides generated after digestion of the molecular mass band above 116 kDa with trypsin showed high similarity with mouse IgG (Table S1).
